# A novel three-dimensional culture system maintaining the physiological extracellular matrix of fibrotic model livers accelerates progression of hepatocellular carcinoma cells

**DOI:** 10.1038/s41598-017-09391-y

**Published:** 2017-08-29

**Authors:** Yuya Miyauchi, Kentaro Yasuchika, Ken Fukumitsu, Takamichi Ishii, Satoshi Ogiso, Takahito Minami, Hidenobu Kojima, Ryoya Yamaoka, Hokahiro Katayama, Takayuki Kawai, Elena Yukie Yoshitoshi-Uebayashi, Sadahiko Kita, Katsutaro Yasuda, Naoya Sasaki, Shinji Uemoto

**Affiliations:** Department of Surgery, Graduate School of Medicine, Kyoto University, Kyoto, Japan

## Abstract

Liver fibrosis is characterized by the progressive accumulation of extracellular matrix (ECM) and is a strong predictor of hepatocellular carcinoma (HCC) development and progression. However, the effect of ECM in fibrotic livers on HCC cells is poorly understood. The aims of this study were to create a new culture system that retained the natural ECM of fibrotic model livers and to establish whether natural ECM regulated the characteristics of HCC cells. Using an organ decellularization technique, we created a new culture system that preserved the tissue-specific ECM of fibrotic model livers from CCl_4_-treated rats. The content of ECM in fibrotic model liver scaffolds was increased and the ECM microstructure was distorted. Quantitative polymerase chain reaction and immunofluorescence assays of HCC cells cultured in fibrotic model liver scaffolds for 7 days showed an epithelial-mesenchymal transition phenotype. Moreover, the ECM of fibrotic model livers promoted proliferation and chemoresistance of HCC cells. These results showed a novel effect of natural ECM in fibrotic model livers on the malignant behaviour of HCC cells. This new culture system will be useful for both understanding the cell biology of fibrotic livers and developing novel anti-cancer drugs.

## Introduction

Liver fibrosis is the primary risk factor for the development and progression of hepatocellular carcinoma (HCC)^1–4^. Fibrosis, caused by chronic injuries to the liver, is characterized by the progressive accumulation of extracellular matrix (ECM). This accumulation distorts the hepatic architecture by forming fibrous bridges and causing mechanical changes in the microenvironment^2^. Several recent reports suggest that the microenvironment of fibrotic livers contributes to HCC progression^5–7^. Reports based on conventional two-dimensional or gel three-dimensional culture systems indicate that the proliferation and chemotherapeutic response of HCC cells is related to increases in matrix stiffness, which is one aspect of the microenvironment in fibrotic livers^5, 6^. However, these culture systems reflect only the matrix stiffness and lack the fine structure of the natural ECM. Effects of the natural ECM, including protein components and microstructures in fibrotic livers, on HCC cells are poorly understood.

Recently, decellularized scaffolds derived from animal organs have been explored as a new platform for examining cell function and differentiation^8–11^, as well as a resource for generating solid organs^12–18^. Decellularized scaffolds provide a surface for cell attachment, room for cell growth and migration as a three-dimension culture system. Moreover, decellularized scaffolds are responsible for promoting the tissue-specific functions and cell differentiation^8–11^. Decellularized scaffolds retain the natural tissue-specific ECM that consists of complex microstructural and functional proteins such as collagen, laminin, fibronectin, and other matrix components^9^. This natural ECM allows cells to maintain their tissue-specific phenotype and this is one of the advantages over conventional two-dimensional and three-dimensional culture systems^8–11^. Kamal, *et al*. reported that HCC cells cultured in decellularized liver scaffolds maintained their invasive ability at a significantly higher level than cells cultured in conventional two-dimensional or gel three-dimensional systems^11^.

The aims of this study were to create a novel *in vitro* culture system that retained the tissue-specific ECM of fibrotic model livers using decellularization technique, and to determine the effect of this fibrotic model liver ECM on the characteristics of HCC cells. We identified the details of ECM obtained from fibrotic model livers. In addition, we demonstrated that the decellularized fibrotic model livers accelerated the epithelial-mesenchymal transition (EMT) phenotype, proliferation and drug resistance of HCC cells. This novel culture system is ideal for studying cancer cell niches in fibrotic livers.

## Results

### Characterization of decellularised normal and fibrotic model liver scaffolds

**Figure 1:**
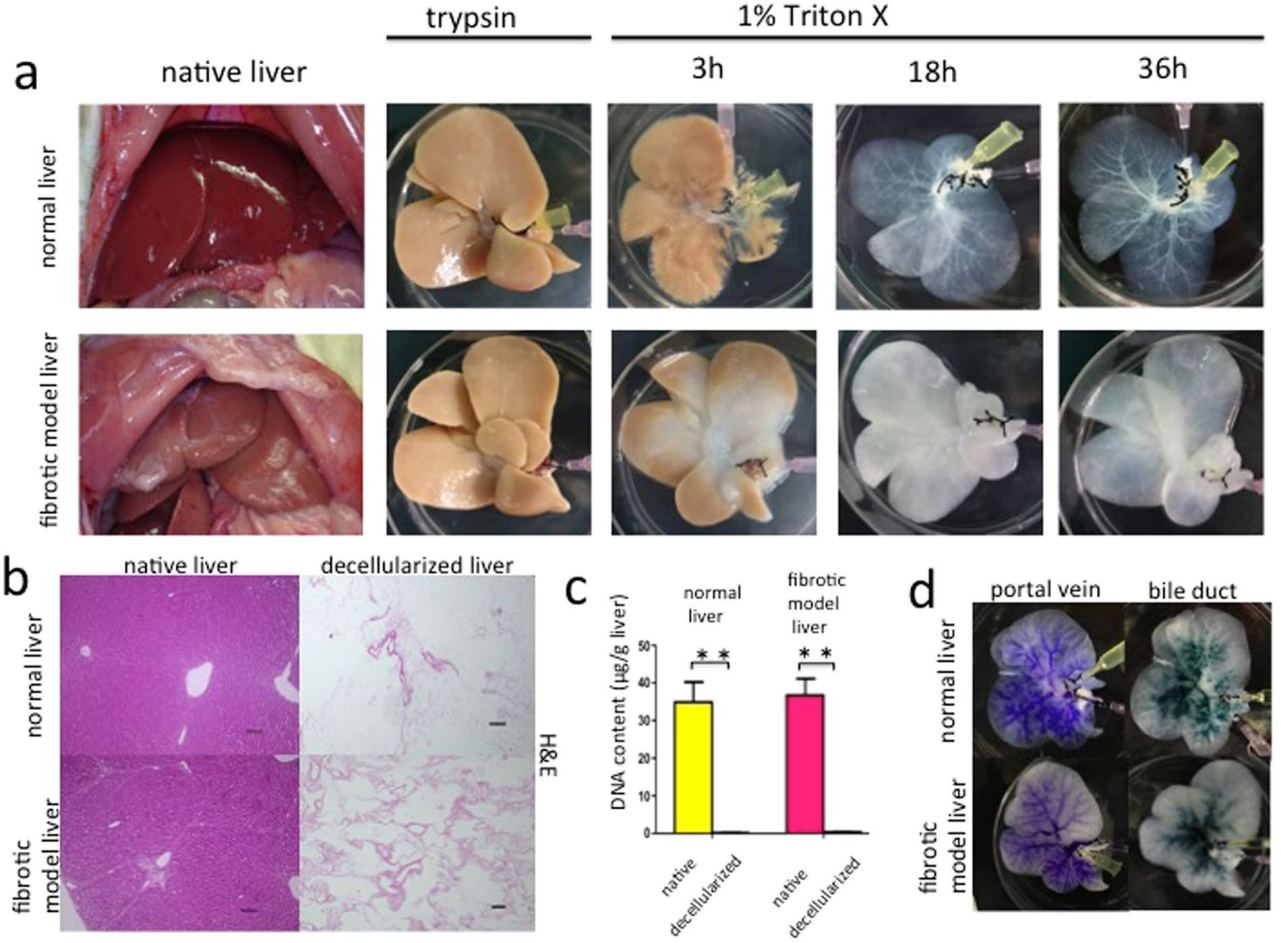
Decellularization of normal and fibrotic model livers. (**a**) Macroscopic appearance of decellularized normal and fibrotic model livers. (**b**) Haematoxylin and eosin staining shows no remaining nuclei and cytoplasm in the scaffolds. (**c**) Quantification of double-stranded DNA shows that the decellularization procedure eliminated more than 99% of the DNA content in native livers. (**d**) Intrahepatic portal vein and bile duct staining demonstrate patency. Data are expressed as means ± SD (n = 3). *p < 0.05 and **p < 0.01 compared to the control. Scale bars: 100 µm.

Translucent white-coloured scaffolds that retained gross anatomical features of native liver were generated after subjecting rat livers to our decellularization procedure. This perfusion procedure required twice as long to achieve complete decellularization in fibrotic model livers than normal livers (Fig. 1a). As we previously reported^17^, decellularised liver scaffolds were evaluated by following methods. Haematoxylin and eosin staining of both the normal and fibrotic model decellularized liver scaffolds revealed the absence of nuclei and cytoplasmic components (Fig. 1b). Measuring residual DNA content in the decellularized liver scaffolds showed that over 99% of the total DNA content was removed (normal livers: native 34.9 ± 5.35 µg/g versus decellularized: 0.15 ± 0.074 µg/g; fibrotic model livers: native 36.7 ± 7.55 µg/g versus decellularized: 0.34 ± 0.064 µg/g, p < 0.01) (Fig. 1c).

The perfusion of blue dye through the portal vein and green dye through the bile duct revealed the morphological integrity of intrahepatic capillaries without dye leakage from the surface of the decellularized livers. This suggested preservation of the vascular and biliary trees in both normal and fibrotic model liver scaffolds (Fig. 1d).

### Fibrosis analysis

**Figure 2:**
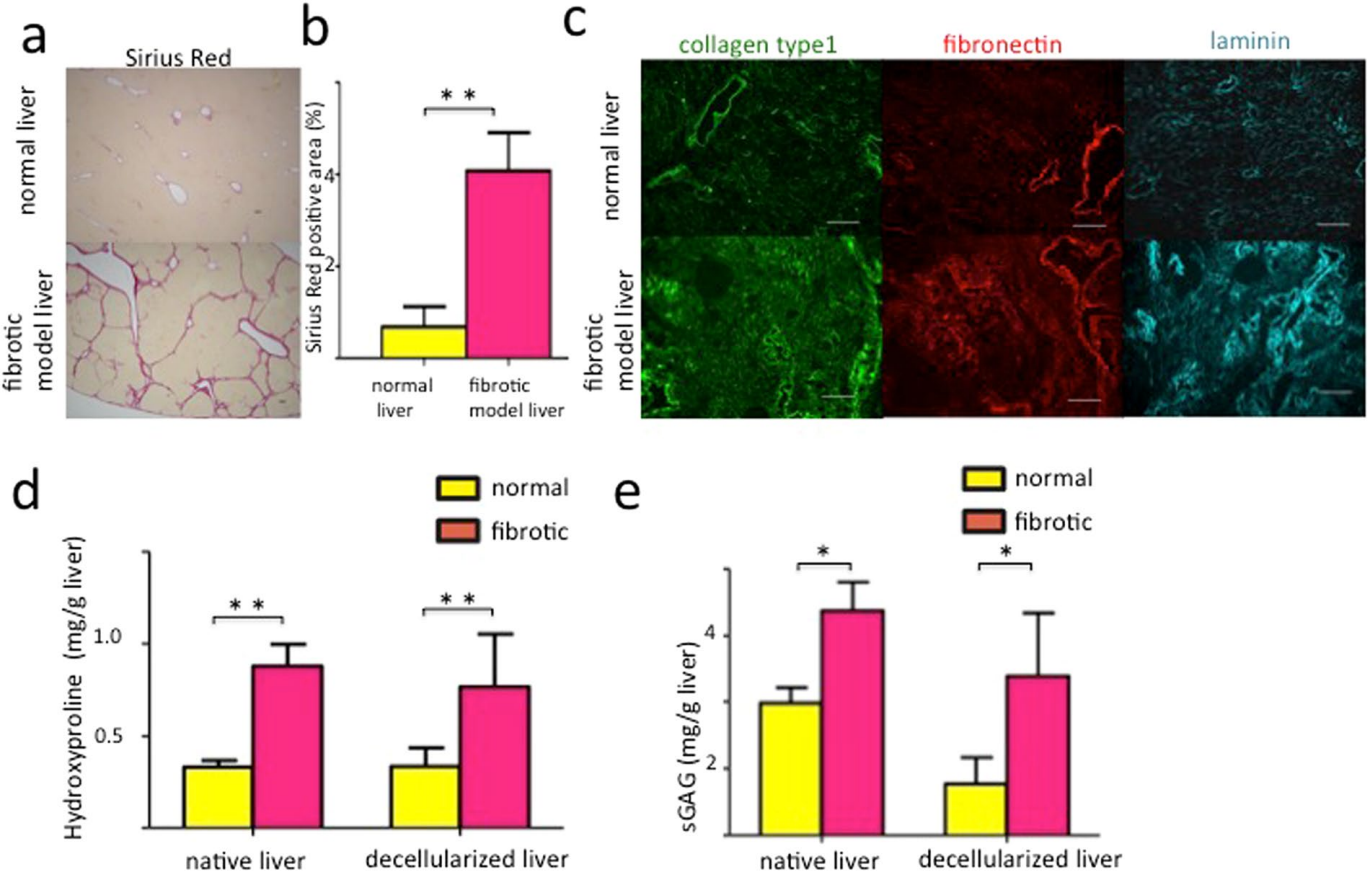
Characterization of decellularized livers. (**a**) Liver fibrosis is evaluated by Sirius Red staining. (**b**) The Sirius Red-positive area is quantified as a percentage of the total area using Image J software. (**c**) Collagen type I, fibronectin and laminin were increased and distributed diffusely in decellularized fibrotic model livers compared to decellularized normal livers. Quantification of extracellular matrix components indicates that hydroxyproline (**d**) and sulphated glycosaminoglycan (sGAG) (**e**) are significantly higher in decellularized fibrotic model livers compared to decellularized normal livers. Data are expressed as means ± SD (n = 3). *p < 0.05 and **p < 0.01 compared to the control. Scale bars: 100 μm.

Fibrotic model rat was induced by repetitive administration of CCl_4_ for 8 weeks. Hepatic collagen deposition was evaluated by Sirius Red staining. Bridging fibrosis between portal and central areas was observed in fibrotic model livers, whereas staining was evident only around the peri-portal region in normal livers (Fig. 2a). The Sirius Red staining positive area was significantly larger in fibrotic model livers compared to that in normal livers (4.08 ± 1.44% versus 0.68 ± 0.44%, p < 0.01) (Fig. 2b).

Subsequently to the appropriate decellurization, we evaluated the fibrosis of decellularized scaffolds by qualitative and quantitative methods. The ECM of decellularized fibrotic model livers was analysed by immunohistochemistry and compared to that of decellularized normal livers. This analysis showed that key ECM components, namely collagen type I, fibronectin and laminin, were increased and disordered in the decellularized fibrotic model livers (Fig. 2c).

The amounts of ECM components were quantified to compare the extent of fibrosis between normal and fibrotic model liver scaffolds. The amount of hydroxyproline in fibrotic model livers increased significantly compared to normal livers, both before and after decellularization (native livers: normal 0.33 ± 0.06 mg/g versus fibrotic model 0.88 ± 0.19 mg/g, p < 0.01; decellularized livers: normal 0.33 ± 0.10 mg/g versus fibrotic model 0.77 ± 0.28 mg/g, p < 0.01) (Fig. 2d). About 90% of hydroxyproline was retained after decellularization in both normal and fibrotic model livers. The amount of sulphated glycosaminoglycan (sGAG) in fibrotic model livers increased significantly compared to normal liver, both before and after decellularization (native livers: normal 1.98 ± 0.41 mg/g versus fibrotic model 3.37 ± 0.75 mg/g, p = 0.024; decellularized livers: normal 0.77 ± 0.39 mg/g versus fibrotic model 2.39 ± 0.94 mg/g, p = 0.026) (Fig. 2e). Approximately 39% of sGAG was retained in normal livers after decellularization and 70% was retained in fibrotic model livers.

### HCC cell cultures

**Figure 3:**
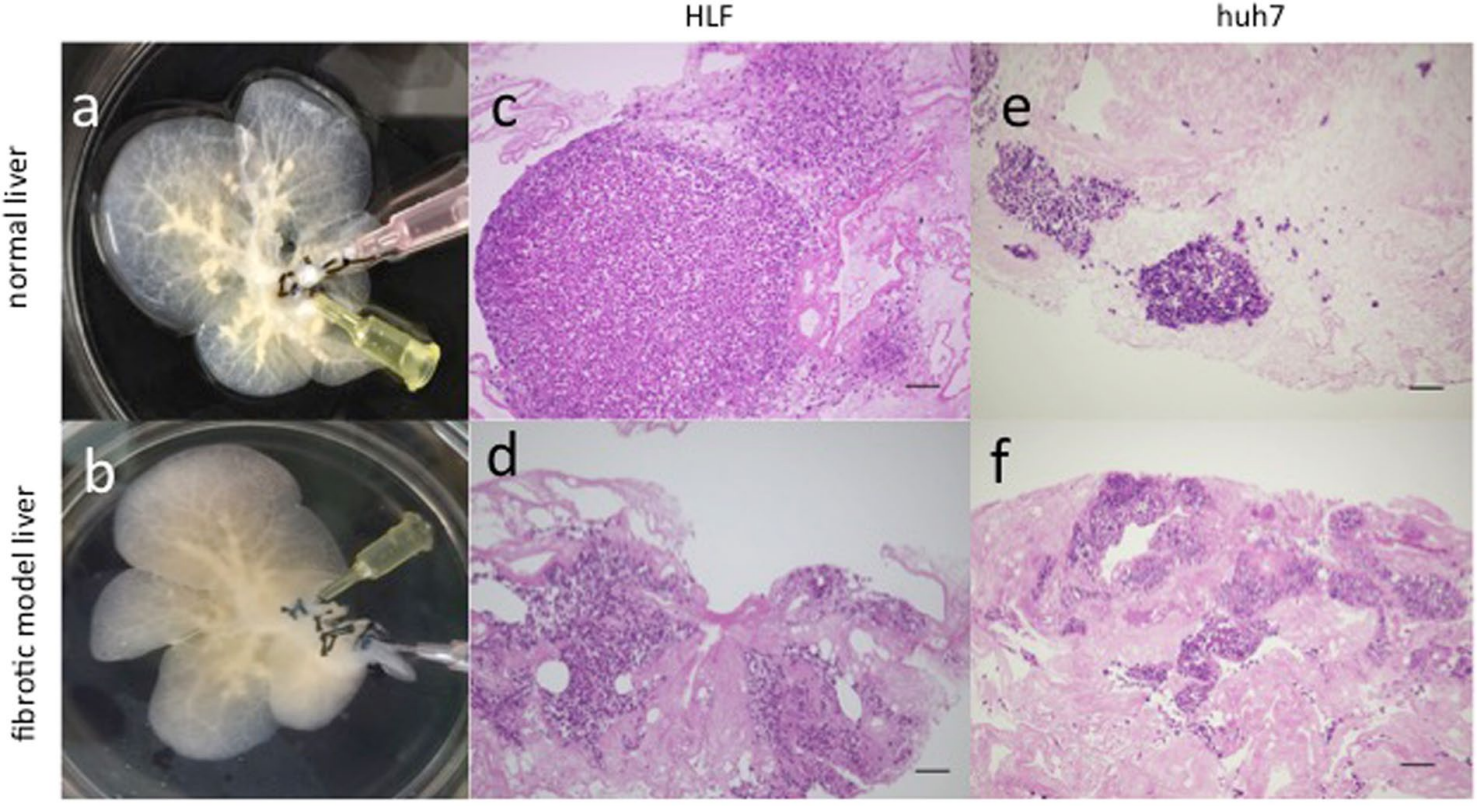
Hepatocellular carcinoma cells cultured in decellularized normal and fibrotic model livers. Macroscopic appearance of HLF cells seeded into decellularized normal (**a**) and fibrotic model (**b**) livers. Histological examination shows that HLF cells are engrafted in decellularized normal (**c**) and fibrotic model (**d**) livers. Huh7 cells in decellularized normal (**e**) and fibrotic model (**f**) livers and are widely distributed in the parenchymal region. Scale bars: 100 μm.

To evaluate the availability of decellularized liver scaffolds as a novel three-dimensional culture system, HLF and Huh7 cells were seeded through the bile duct into normal (Fig. 3a) and fibrotic model (Fig. 3b) liver scaffolds. Seven days after seeding, histological evaluation revealed that HCC cells were engrafted into the liver parenchymal space. HCC cells in normal liver scaffolds progressed to forming nodules (Fig. 3c: HLF cells; Fig. 3e: Huh7 cells), whereas HCC cells in fibrotic model liver scaffolds progressed with an infiltrating pattern among the thick and disordered ECM (Fig. 3d: HLF cells; Fig. 3f: Huh7 cells).

### HCC cells in fibrotic model liver scaffolds acquired the EMT phenotype

**Figure 4:**
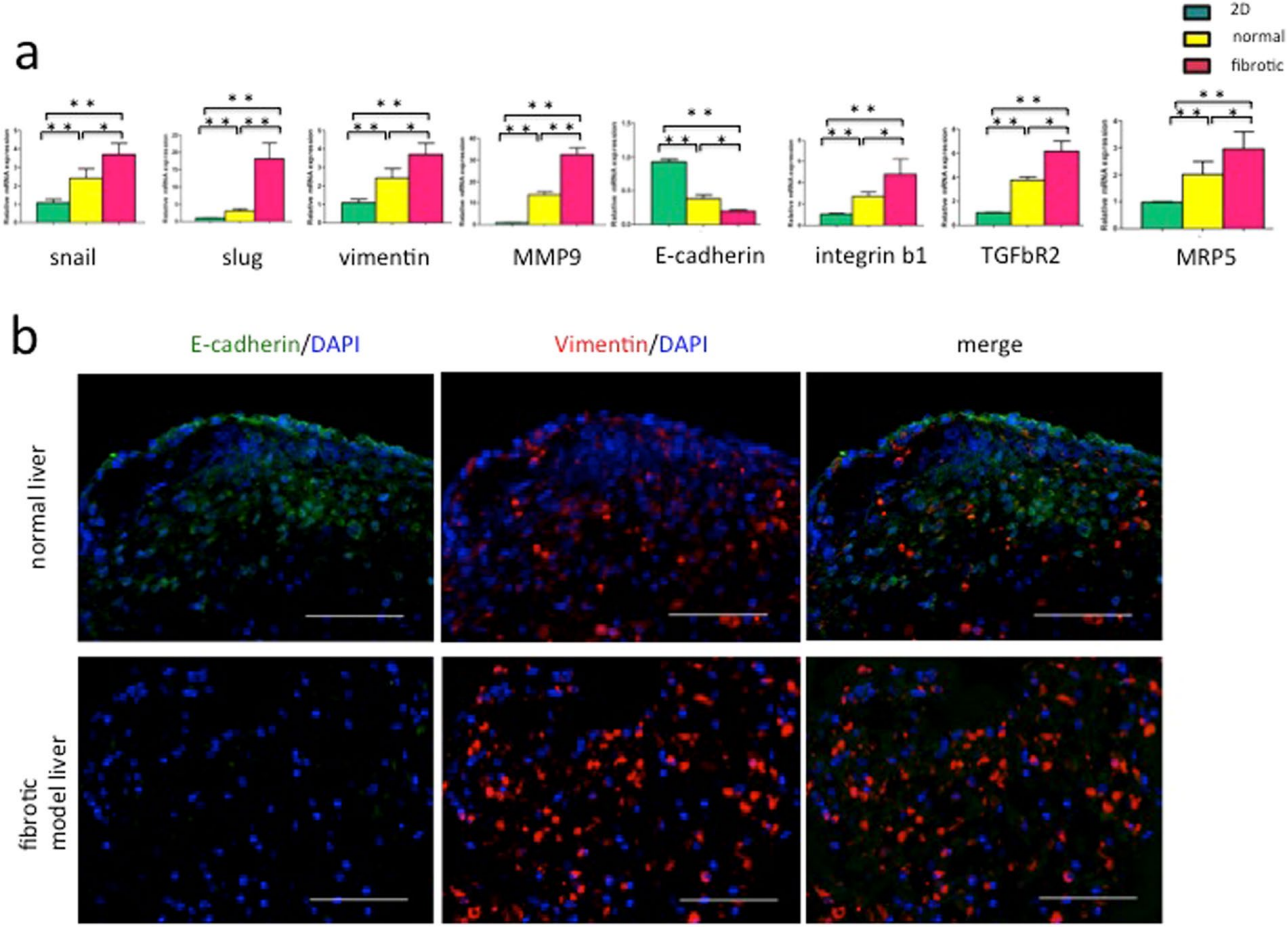
Gene expression and epithelial-mesenchymal transition phenotype analyses. (**a**) Normalized gene expression of Snail, Slug, vimentin, MMP-9, E-cadherin, MRP5, integrin β1 and TGFβR2 mRNA in HLF cells is measured by qRT-PCR. Gene abbreviations: MMP-9, matrix metalloproteinase-9; MRP5, multidrug resistance protein 5; TGFβR2, Transforming growth factor beta receptor 2. (**b**) Immunofluorescence staining shows that the number of vimentin-positive cells is increased and the number of E-cadherin-positive cells is decreased in fibrotic model liver scaffolds compared to normal liver scaffolds. This indicates that fibrotic model liver scaffolds promote the EMT phenotype. Scale bars: 100 μm. Data are expressed as means ± SD (n = 3). *p < 0.05 and **p < 0.01 compared to controls.

To investigate the effect of fibrotic model liver-derived ECM on HCC cells, gene expression was analysed in HLF and Huh7 cells by the quantitative reverse transcription polymerase chain reaction (qRT-PCR). Cells were cultured in a conventional two-dimensional culture, normal liver scaffolds and fibrotic model liver scaffolds for 1 and 7 days. There was no significant difference in the gene expressions of HCC cells among these three culture systems at day1 (HLF cells: Supplemental Fig. 1A; huh7 cells: Supplemental Fig. 1B). At day 7, the expression of mesenchymal markers, such as Snail, Slug, vimentin, and matrix metalloproteinase-9 (MMP-9) was significantly higher in three-dimensional scaffolds compared to two-dimensional culture (HLF cells: Fig. 4a; huh7 cells: supplemental Fig. 2). In addition, these markers were significantly up-regulated in fibrotic model liver scaffolds compared to normal liver scaffolds. In contrast, expression of the epithelial marker, E-cadherin, was significantly down-regulated in HCC cells cultured in fibrotic model liver scaffolds compared to normal liver scaffolds and two-dimensional culture.

Immunofluorescence staining showed that the number of vimentin-positive cells was increased in fibrotic model liver scaffolds compared to normal liver scaffolds, whereas the number of E-cadherin-positive cells was decreased (Fig. 4b).

### HCC cells in fibrotic model liver scaffolds had higher proliferation ability

**Figure 5:**
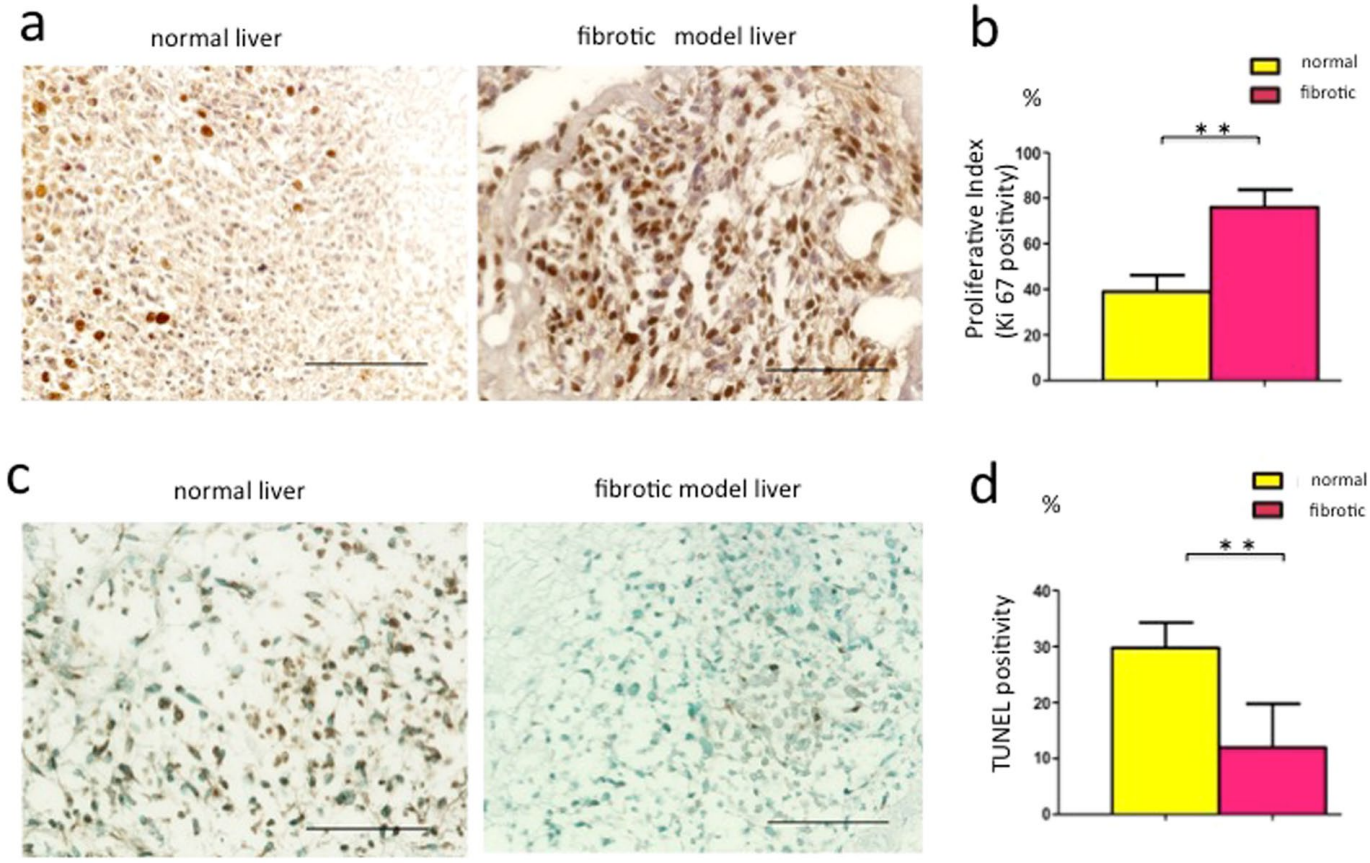
Proliferation and chemoresistance of hepatocellular carcinoma cells in normal and fibrotic model liver scaffolds. (**a**) Quantification of HLF cell proliferation at day 7 using anti-Ki67 staining in decellularized normal and fibrotic model liver scaffolds. (**b**) Graphs showing the mean proliferative index (Ki67 positivity) of HLF cells in decellularized normal and fibrotic model liver scaffolds. (**c**) Quantification of apoptosis by TUNEL staining in normal and fibrotic model liver scaffolds. (**d**) Significantly more cells are TUNEL-positive in normal liver scaffolds than in fibrotic model liver scaffolds. Scale bars: 100 μm. Data are expressed as means ± SD (n = 3). *p < 0.05 and **p < 0.01 compare﻿d to controls.

To assess the proliferation of HLF cells in normal and fibrotic model liver scaffolds, Ki67 staining was performed after 7 days in culture (Fig. 5a). The results demonstrated that a higher proportion of cells were Ki67-positive in fibrotic model than normal liver scaffolds (39.0 ± 7.2% in normal versus 76.0 ± 13.1% in fibrotic model, p < 0.01) (Fig. 5b).

### HCC cells in fibrotic model liver scaffolds had greater resistance to 5-fluorouracil (5-FU)

HCC cells have the ability to resist conventional chemotherapeutic agents. We investigated whether fibrotic model liver scaffolds regulated the susceptibility of HCC cells to the anti-cancer drug, 5-FU. The qRT-PCR showed that the level of multi-drug resistance-associated protein 5 (MRP5) mRNA was significantly up-regulated in fibrotic model liver scaffolds compared to normal liver scaffolds (Fig. 4a). 5-FU-induced apoptosis of HCC cells was assessed using terminal deoxynucleotidyl transferase dUTP nick end labelling (TUNEL) staining. After treatment with 0.1 µM 5-FU, the proportion of TUNEL-positive HCC cells cultured in fibrotic model liver scaffolds (12.0 ± 7.8%) was significantly lower than that in normal liver scaffolds (29.8 ± 7.8%) (p < 0.05) (Fig. 5c,d).

### Integrin β1 and phosphorylated focal adhesion kinase (pFAK) were overexpressed in HCC cells in fibrotic model liver scaffolds

**Figure 6:**
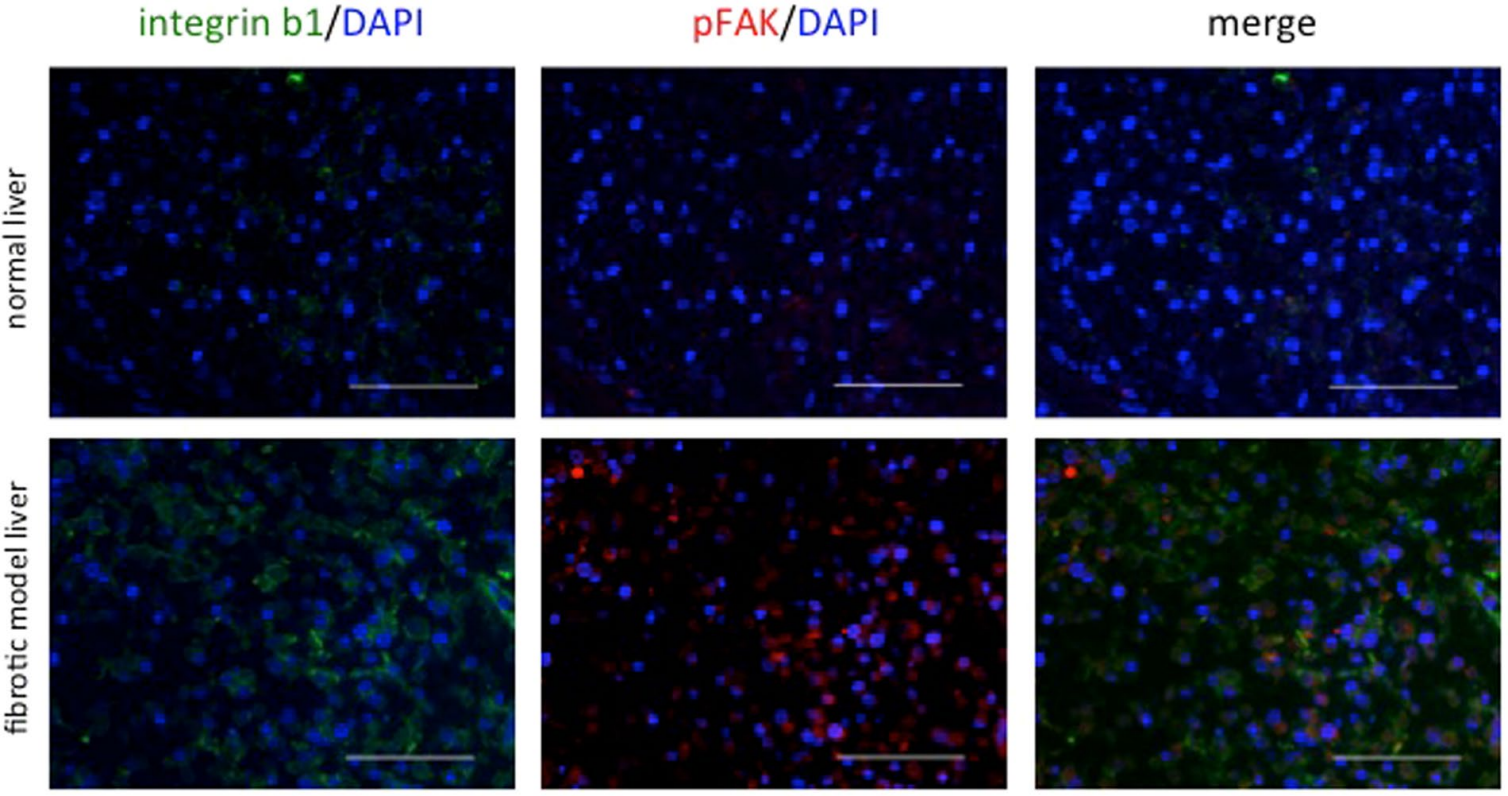
Integrin β1 and phosphorylated focal adhesion kinase (pFAK) expression in HLF cells in decellularized liver scaffolds. Immunofluorescence staining shows that integrin β1 and pFAK expression is up-regulated to a greater extent in fibrotic model liver scaffolds compared to that in normal liver scaffolds. Scale bars: 100 μm.

To demonstrate the underlying mechanism of accelerating EMT phenotype, proliferation and chemoresistance of HCC cells in fibrotic model liver scaffolds, we evaluated the expression of integrins and FAK. The qRT-PCR assay showed that the expression of integrin β1 and transforming growth factor beta receptor 2 (TGFβR2) mRNA was significantly higher in HCC cells cultured in fibrotic model liver scaffolds compared to normal liver scaffolds (Fig. 4a). Additionally, we used immunofluorescence to investigate the expression of integrin β1 and pFAK proteins in HLF cells in normal and fibrotic model liver scaffolds (Fig. 6). Integrin β1 was strongly overexpressed in HLF cells in fibrotic model liver scaffolds. pFAK in HLF cells was also increased in fibrotic model liver scaffolds.

## Discussion

In this study, we created a novel culture system that retained the natural ECM of fibrotic model livers using an established decellularization technique^12, 13, 15, 17^. HCC cells infused via the bile duct were distributed effectively into the parenchymal region and cultured successfully for 7 days.

These decellularized fibrotic model liver scaffolds preserved natural physiological ECM and retained intact vascular networks while efficiently removing the cellular and nuclear components. The amounts of ECM components in fibrotic model livers were significantly increased compared to those in normal livers both before and after decellularization. Immunohistochemistry revealed that the ECM microstructure in fibrotic model livers scaffolds was distorted. These results suggested that this novel culture system mimicked the altered microenvironment of fibrotic livers.

The EMT is a major mediator of tumour metastasis and recurrence^19, 20^. During the process of metastasis, tumour cells acquire mesenchymal properties, enabling them to migrate through and invade surrounding tissues, and even enter the bloodstream. This leads to invasion, metastasis, and recurrences. In this study HCC cells in fibrotic model liver scaffolds grew in an infiltrative pattern, whereas HCC cells in normal liver scaffolds progressed to forming nodules. The analysis of qRT-PCR and immunofluorescence showed that HCC cells cultured in fibrotic model liver scaffolds acquired the mesenchymal characteristics. These results indicated that the natural ECM of fibrotic model livers profoundly accelerated the EMT phenotype of HCC cells and that the infiltrative tumour-forming pattern in fibrotic model liver scaffolds was derived from EMT-related changes. In addition, Ki-67 and TUNEL staining suggested that fibrotic model liver scaffolds promoted higher proliferation ability and greater drug resistance of HCC cells than normal liver scaffolds. Overall, these results showed the novel effect of a physiological ECM in fibrotic model livers on the malignant behaviour of HCC cells.

Integrins are major cell surface receptors for ECM and FAK is a key downstream effector of integrins. Integrin-FAK signaling plays essential roles in a variety of biological processes. Integrin-FAK signaling activates the mitogen-activated protein kinase (MAPK)/extracellular signal-regulated protein kinase (ERK) cascade to promote cell growth, whereas the phosphatidylinositol 3-kinase (PI3K) pathway initiated by integrin-FAK signaling prevents apoptosis^21^. Moreover, several studies revealed the crosstalk between integrin and TGF-β, one of the most potent inducers of the EMT^21–23^. In our study, the expression of integrin β1 and pFAK was up-regulated to a greater extent in HCC cells cultured in fibrotic model liver scaffolds compared to that in normal liver scaffolds. These results are consistent with previous reports^5, 6, 22, 23^ and support the hypothesis that altered ECM in fibrotic model livers promotes the TGF-β mediated EMT phenotype, cell proliferation and drug resistance through integrin-FAK signalling.

There are several reports that decellularized scaffolds are useful as a drug testing system^11, 24^. The natural ECM allows HCC cells to sustain the abilities of drug metabolism and proliferation^11^. Therefore, HCC cells cultured in decellularized scaffolds can be used to evaluate drug efficacy more accurately than in conventional culture systems^11^. Furthermore, our results demonstrate that HCC cells cultured in fibrotic model liver scaffolds have greater resistance and proliferations than either two-dimensional culture or normal liver scaffolds. Approximately 70–90% of patients with HCC have an established background of chronic liver disease and cirrhosis^3^. Therefore, fibrotic model liver scaffolds would be appropriate to drug discovery of HCC.

This study has several potential limitations. First, it was difficult to observe HCC cells in these decellularized liver scaffolds over time during cultivation. As a result, morphological changes and viability of HCC cells were only assessed histologically. Second, the effect of fibrotic model liver scaffolds on HCC cells was not evaluated directly. There still remains a possibility that the environment in fibrotic model liver scaffolds itself selected the specific cells that already have malignant potential, irrespective of cell-to-ECM attachment. Third, this culture system mimicked only the ECM of fibrotic livers. The microenvironment of HCC is composed of cellular elements, cytokines and growth factors other than ECM. Thus, studies including additional cellular elements, such as cancer-associated fibroblasts and hepatic stellate cells, are required to investigate the behavior of HCC cells under more *in vivo*-like conditions.

In conclusion, the current study describes a novel three-dimensional *in vitro* culture system that mimics the microenvironment of fibrotic livers. This is the first report that the natural ECM of fibrotic model livers can be retained and used as a culture system. This new culture system promotes the EMT phenotype and increases proliferation and chemoresistance in HCC cells. Thus, it will be useful to investigate the biological behaviour of HCC cells in fibrotic livers, and to evaluate and predict the efficacy of new anti-cancer drugs.

## Methods

### HCC cell lines

The human HCC cell lines, Huh7 and HLF, were cultured at 37 °C under 5% CO_2_ in Roswell Park Memorial Institute 1640 medium (RPMI 1640; Invitrogen, Carlsbad, CA, USA) supplemented with 10% foetal bovine serum (ICN, Aurora, OH, USA), 100 U/mL penicillin G, and 100 μg/mL streptomycin (Meiji Seika, Tokyo, Japan). Cells were passaged every 7–10 days. Cell suspensions were obtained from confluent culture dishes using a trypsin solution followed by determining the cell number using a haemocytometer.

### Fibrotic model liver

Male Lewis rats (8 weeks old) were used for establishing the liver fibrosis model. The rats were injected intraperitoneally with CCl_4_ (l mL/kg body weight; 1:1 dilution with corn oil) twice a week for 8 weeks. Sixteen-week-old male rats were used as normal controls. All experimental protocols were approved by the Animal Experimentation Committee of Kyoto University and all animal experimental procedures were performed according to the Animal Protection Guidelines of Kyoto University.

### Liver harvest

Livers were harvested using a technique described previously^14^. Briefly, rats were anesthetized with isoflurane and underwent laparotomy. Heparin (0.5 U/g body weight) was administered intravenously. The abdominal aorta was cannulated with a 24-gauge cannula, and 50 mL of phosphate-buffered saline (PBS) was injected. The liver was removed, followed by the cannulation of the portal vein with an 18-gauge cannula, and the bile duct with a 24-gauge cannula. The livers were stored in cell culture dishes with PBS solution and frozen at −80 °C until decellularization.

### Preparation of decellularized liver scaffolds

Harvested normal and fibrotic model livers were decellularized according to the protocol described previously^14^. Briefly, frozen livers were thawed at room temperature. This was followed by cell-ECM detachment using perfusion with a 0.02% trypsin/0.05% EGTA solution through the portal vein for 1 h at 37 °C. Subsequently, a 1% Triton X-100/0.05% EGTA solution was perfused at 1 mL/min until all cells were removed. Samples were washed with PBS for 1 h. Then, the decellularized liver scaffold was perfused with 0.1% peracetic acid for 2 h for sterilization.

### Vascular tree imaging

The patency of the intrahepatic vascular system was evaluated by perfusion with a blue dye through the portal vein and a green dye through the bile duct, using a peristaltic pump.

### Recellularization and static culture

Decellularized liver scaffolds were placed in 60-mm culture dishes and perfused with RPMI 1640 medium through the portal vein prior to recellularization. RPMI 1640 was supplemented with 10% foetal calf serum, 100 U/mL penicillin G, 100 μg/mL streptomycin, and 1 µg/mL amphotericin B (Wako Pure Chemical Industries, Osaka, Japan). HCC cells were re-suspended at a concentration of 30 million cells per 30 mL RPMI 1640 medium per scaffold, and seeded into the scaffold through the bile duct manually over 30 min. After culturing for 3 h in an incubator, recellularized liver grafts were cut into small pieces (about 10 mm). The liver grafts were cultured in an incubator for 7 days. Culture medium was changed every 48 h.

### Determination of DNA, collagen, and sGAG contents

DNA content was measured using the Qubit dsDNA HS Assay Kit (Invitrogen) to ensure the complete removal of native cells from the liver scaffolds according to the manufacturer’s protocol. To compare the amount of ECM components in normal and fibrotic model rat liver scaffolds, the collagen and sGAG contents were measured. The collagen content was measured indirectly through measurements of hydroxyproline as described previously^25^. sGAG was determined using the Blyscan Assay (Biocolor, Newtownabbey, UK) according to the manufacturer’s instructions. All data from decellularized livers were normalized to the initial liver weight.

### Histology and immunohistochemistry

Decellularized liver scaffolds and recellularized liver grafts were fixed with 4% paraformaldehyde, embedded in paraffin, and cut into 5-μm thick sections. Hematoxylin and eosin, and Sirius Red staining were performed according to standard protocols. Five fields at high magnification were selected randomly and photographed. The Sirius Red-positive area was quantified using Image J software (National Institutes of Health, Bethesda, MD, USA) as reported previously^26^.

Immunofluorescence analyses were performed as reported previously^25^. Antigen retrieval was achieved with a Target Retrieval Solution (Dako). Nonspecific binding was blocked with 1.4% bovine serum albumin (Sigma-Aldrich, St. Louis, MO, USA) dissolved in 0.1% saponin (Wako Pure Chemical Industries) in PBS. Sections were incubated overnight at 4 °C with the following primary antibodies: rabbit polyclonal anti-collagen 1 (1:200; Bioss Antibodies, Woburn, MA, USA), rabbit polyclonal anti-fibronectin (1:200; Bioss Antibodies), rabbit polyclonal anti-laminin (1:200; Bioss Antibodies), rabbit polyclonal anti-E-cadherin (1:50; Cell Signaling Technology, Beverly, MA, USA), mouse polyclonal anti-vimentin (1:200; Abcam, Cambridge, UK), mouse polyclonal anti-integrin β1 (1:200; BD Biosciences, San Jose, CA, USA), and rabbit polyclonal anti-pFAK (1:50; Abcam). After washing, the stained sections were incubated with Alexa 488-conjugated donkey anti-rabbit IgG (1:500; Invitrogen), Alexa 488-conjugated goat anti-mouse IgG (1:500; Invitrogen), Alexa 555-conjugated donkey anti-rabbit IgG (1:500; Abcam), or Alexa 555-conjugated goat anti-mouse IgG (1:500; Invitrogen) for 2 h at room temperature. After washing, the stained sections were covered with Vectashield mounting medium containing 4,6-diamidino-2-phenylindole (Vector Laboratories, Burlingame, CA, USA). All samples were imaged using a BZ-9000 fluorescence microscope (Keyence, Osaka, Japan).

Ki67 expression in the engrafted cells was analysed immunohistochemically to assess their proliferative capacity. Deparaffinized sections were incubated in 3% hydrogen peroxide for 10 min to block endogenous peroxidase activity. Sections were then incubated at 4 °C for 16 h with anti-human Ki67 mouse antibodies (BioLegend, San Diego, USA) diluted at 1:200. After the primary antibody was washed off, samples were incubated for 60 min with horseradish peroxidase-conjugated anti-mouse IgG (Envision Plus Kit; Dako, Glostrup, Denmark) and for 1 min with 3,3-diaminobenzidine substrate (Dako). Nuclei were counterstained with haematoxylin. The cellular proliferative index (Ki67-positive cells/total cells) was calculated by direct cell counting of five randomly selected high magnification photomicrographs from Ki67-stained slides (n = 3).

### Reagents and drug resistance assay

5-FU was purchased from Wako Pure Chemical Industries and dissolved in RPMI 1640. After 48 h incubation for recellularization of the scaffolds with HLF cells, the reseeded specimens were cultured with medium containing 0.1 µM 5-FU for 48 h, followed by further incubation for 48 h. The resistance of cells to 5-FU was assessed using the TUNEL assay. TUNEL staining was performed using an *In situ* Apoptosis Detection Kit (Takara Bio, Shiga, Japan) according to the manufacturer’s protocol. Samples were incubated for 1 min with 3,3-diaminobenzidine substrate and counterstained with methyl green. The TUNEL-positive cell index was calculated by direct cell counting of three randomly selected high magnification photomicrographs from Ki67 stained slides (n = 3).

### qRT-PCR

Total RNA was extracted using a PureLink RNA Mini Kit (Invitrogen). Total RNA (500 ng) was reverse transcribed into cDNA using a ReverTra Ace kit (Toyobo, Osaka, Japan) according to the manufacturer’s instructions. Primers for the amplification of Snail, Slug, vimentin, E-cadherin, matrix metalloproteinase-9, MRP5, integrin β1, TGFβR2 and β-actin were used (Supplementary Table S1). The qRT-PCR assay was performed using SYBR-green PCR Master Mix (Applied Biosystems, Foster City, CA, USA) on an ABI 7500 system (Applied Biosystems). Each experiment was performed in duplicate. mRNA expression levels were normalized to the levels of β-actin.

### Statistical analyses

Data are expressed as means ± standard deviation. Statistical analyses were performed using GraphPad Prism for Windows, version 6.0 (GraphPad Software, La Jolla, CA, USA). Statistical significance was defined as p < 0.05. DNA, hydroxyproline, and sGAG contents, mRNA expression levels, and the cell proliferation index were compared using Student’s t-test.

## Electronic supplementary material

Supplementary Information
