# Supplementary material for: A novel three-dimensional culture system maintaining the physiological extracellular matrix of fibrotic model livers accelerates progression of hepatocellular carcinoma cells
Source: Sci Rep. 2017 Aug 29;7:9827. doi: 10.1038/s41598-017-09391-y (PMC5575302; doi:10.1038/s41598-017-09391-y)

- **A novel three-dimensional culture system maintaining the physiological extracellular matrix of fibrotic model livers accelerates progression of hepatocellular carcinoma cells**
- Yuya Miyauchi, Kentaro Yasuchika^*^, Ken Fukumitsu, Takamichi Ishii, Satoshi Ogiso, Takahito Minami, Hidenobu Kojima, Ryoya Yamaoka, Hokahiro Katayama, Takayuki Kawai, Elena Yukie Yoshitoshi, Sadahiko Kita, Katsutaro Yasuda, Naoya Sasaki, Shinji Uemoto
- Department of Surgery, Graduate School of Medicine, Kyoto University, Kyoto, Japan

*Contact information:

Kentaro Yasuchika, M.D., Ph.D.

Department of Surgery, Graduate School of Medicine, Kyoto University, 54 Kawara-cho, Shogoin, Sakyo-ku, Kyoto, 606-8507, Japan.

E-mail: kent@kuhp.kyoto-u.ac.jp

Tel: +81-75-751-3242; Fax: +81-75-751-4246


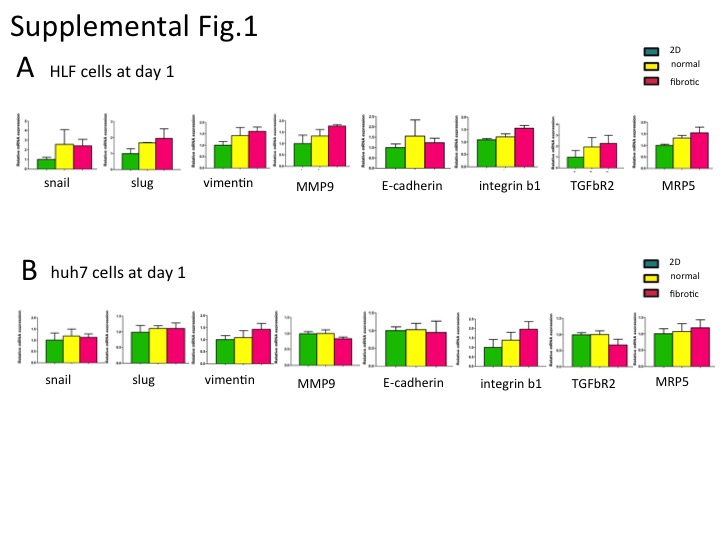


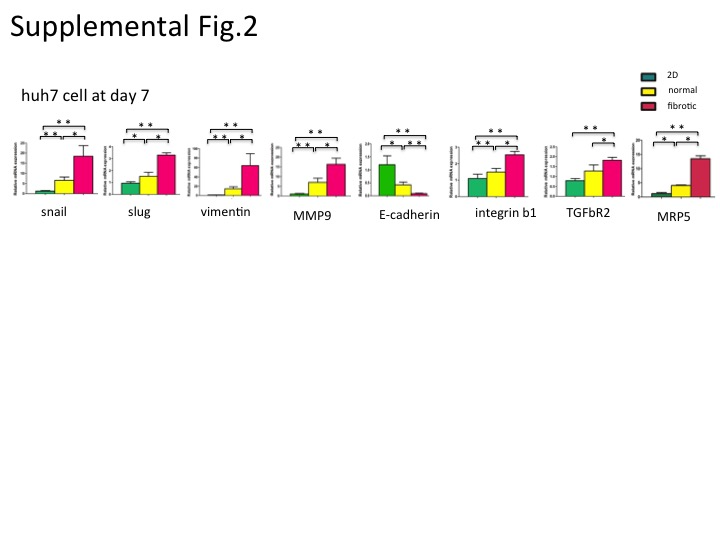


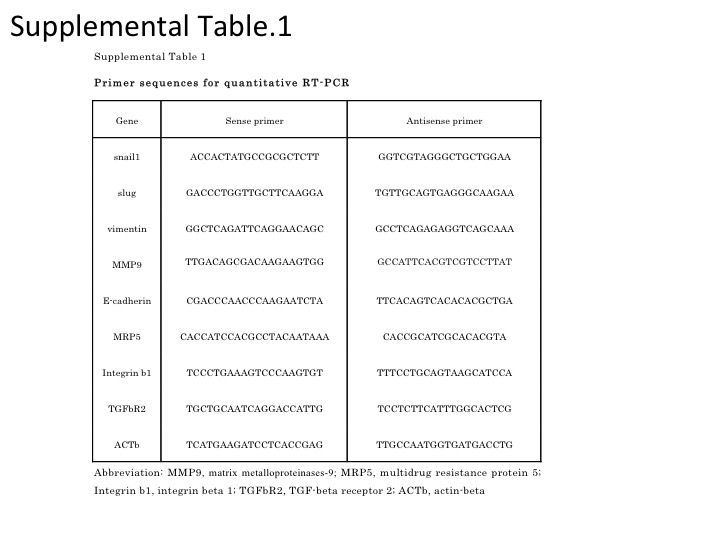

Supplement: Supplementary file 1 — Supplementary Information [file 41598_2017_9391_MOESM1_ESM.doc]
